# Artificial Intelligence in Drug Metabolism and Excretion Prediction: Recent Advances, Challenges, and Future Perspectives

**DOI:** 10.3390/pharmaceutics15041260

**Published:** 2023-04-17

**Authors:** Thi Tuyet Van Tran, Hilal Tayara, Kil To Chong

**Affiliations:** 1 Department of Electronics and Information Engineering, Jeonbuk National University, Jeonju 54896, Republic of Korea; tttvan@jbnu.ac.kr; 2 Faculty of Information Technology, An Giang University, Long Xuyen 880000, Vietnam; 3 Vietnam National University—Ho Chi Minh City, Ho Chi Minh 700000, Vietnam; 4 School of International Engineering and Science, Jeonbuk National University, Jeonju 54896, Republic of Korea; 5 Advances Electronics and Information Research Center, Jeonbuk National University, Jeonju 54896, Republic of Korea

**Keywords:** drug discovery, drug metabolism, drug excretion, artificial intelligence, machine learning, deep learning, in silico method, web servers

## Abstract

Drug metabolism and excretion play crucial roles in determining the efficacy and safety of drug candidates, and predicting these processes is an essential part of drug discovery and development. In recent years, artificial intelligence (AI) has emerged as a powerful tool for predicting drug metabolism and excretion, offering the potential to speed up drug development and improve clinical success rates. This review highlights recent advances in AI-based drug metabolism and excretion prediction, including deep learning and machine learning algorithms. We provide a list of public data sources and free prediction tools for the research community. We also discuss the challenges associated with the development of AI models for drug metabolism and excretion prediction and explore future perspectives in the field. We hope this will be a helpful resource for anyone who is researching in silico drug metabolism, excretion, and pharmacokinetic properties.

## 1. Introduction

**Figure 1:**
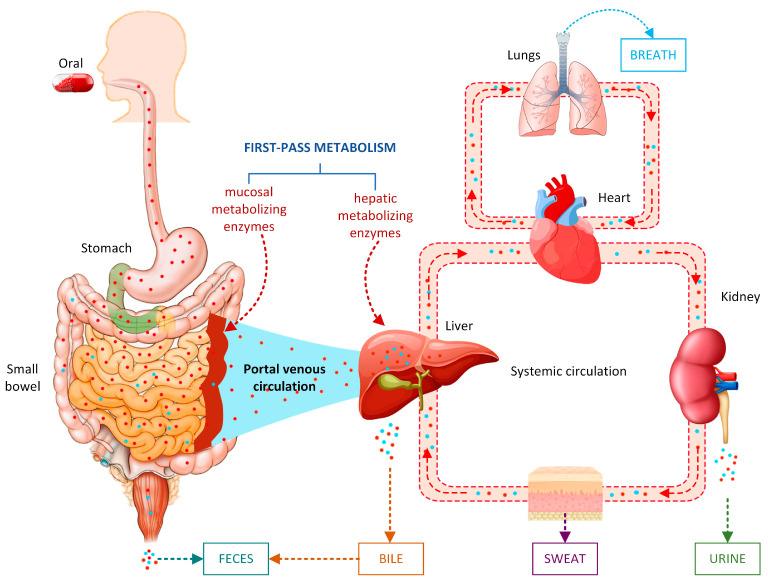
Overview of drug metabolism and excretion.

Metabolism and excretion are two important processes in pharmacokinetics. Figure 1 shows an overview of drug metabolism and excretion [1,2]. Metabolism is the biological transformation by which most drugs undergo a change in their chemical structure in the body to produce the expected therapeutic effects of a certain drug and be more easily eliminated from the body [3]. Drug excretion refers to the elimination of drugs or their metabolites from the body [4]. Drug metabolism can yield metabolites that differ greatly from the original drug’s physical and pharmacological characteristics [5]. The rate of metabolism dictates the length and strength of a drug’s pharmacologic effect. Drug metabolism also plays a role in multidrug resistance in infectious illnesses and cancer chemotherapy, and the effects of certain medications as inhibitors or substrates of enzymes involved in xenobiotic metabolism are frequent causes of adverse drug interactions [6]. Drug metabolism affects drug efficacy and toxicity in humans and laboratory animals. Metabolism is also responsible for the clearance of more than 70% of clinical medicines [5,7], so it has been extensively researched as part of drug research and development (R&D) efforts. Both metabolism and excretion are tightly regulated by the body to maintain homeostasis and ensure that harmful substances are eliminated from the body. Disruptions to these processes can lead to the accumulation of toxic substances, which can cause many health problems, such as kidney and liver damage, metabolic disorders, and drug toxicity [8]. Drug metabolism and excretion play critical roles in the pharmacokinetics of drugs and have important implications for the R&D of new drugs, as well as for the safe and effective use of existing drugs. By understanding and predicting drug metabolism and excretion, researchers can screen unwanted drug candidates and design new drugs with improved pharmacokinetics, reduced toxicity, and increased efficacy.

Predicting drug metabolism and excretion by in vitro and in vivo research is one strategy. These experimental assessments of metabolism and excretion are typically time- and money-consuming. For instance, testing a CYP inhibition from a non-good laboratory practice costs about USD 1000 and takes one week [9]. Given the high expense of conventional drug R&D, numerous computational algorithms for predicting the metabolism and excretion of therapeutic candidates have been developed, allowing for the screening of a large number of chemical compounds and subsequently finding a small number of viable candidates [10]. Especially, in silico approaches are increasingly being used to predict drug metabolism and excretion, and are widely regarded as the best “fail early and fail cheap strategy”, allowing for lower costs, time savings, and thus lower attrition rates in the late stages of drug development.

Artificial intelligence (AI) can now be employed across the entire process of developing new medicines [11]. AI methods are also increasingly being used in the field of drug metabolism and excretion to predict the potential of drugs to be metabolized and excreted by the body. The use of AI allows for the rapid screening of vast libraries of compounds, yielding useful insights into the compounds’ potential metabolism and excretion. Predictions made using AI techniques may be more accurate than those made using more conventional approaches since they can be trained using enormous amounts of experimental data. Predicting the likelihood of metabolic and excretory interactions between numerous drugs at once is a strength of AI systems that can aid in drug discovery. AI techniques can provide useful information on the potential for metabolism and excretion, reducing the time and money needed to conduct in vitro and in vivo experiments. Moreover, the use of AI techniques in drug R&D has the potential to increase both the safety and effectiveness of drugs by creating new compounds with enhanced metabolism and excretion. In this review, we have summarized the background of drug metabolism and excretion and highlighted the key properties of these processes. We took a deep dive into the most recent developments in the use of AI for medication metabolism and excretion prediction. We also provide the research community with a directory of publicly available resources for predicting metabolism and excretion. Research in this area has a number of obstacles, yet there is also promising future growth. We hope this review will be of interest to researchers working to enhance and develop several high-precision prediction models for drug metabolism and excretion.

## 2. Evaluation Metrics

Evaluating the performance of AI methods is critical for measuring a method’s effectiveness and fairly comparing the score of various models [12]. In this review, we present the following evaluation metrics: coefficient of determination (R^2^), root mean squared error (RMSE), specificity (SP), sensitivity (SE), Matthew’s correlation coefficient (MCC), precision, recall, F1 score, accuracy (ACC), Jaccard score, and area under the receiver operating characteristic curve (AUC). The formulas are as follows:

(1) $$R^{2}=1-\frac{\sum {\left(y_{i}-\;\hat{y}\right)}^{2}}{\sum {\left(y_{i}-\;\overset{-}{y}\right)}^{2}}\;\mathrm{Range}\;\left[0,1\right]$$

(2) RMSE=1N∑i=1Nyi− y ^2

(3) $$\mathrm{SE}=\frac{\mathrm{TP}}{\mathrm{TP}+\mathrm{FN}}\;\mathrm{Range}\;\left[0,\;1\right]$$

(4) $$\mathrm{SP}=\frac{\mathrm{TN}}{\mathrm{TN}+\mathrm{FP}}\;\mathrm{Range}\;\left[0,\;1\right]$$

(5) $$\mathrm{MCC}=\frac{\left(\mathrm{TP}*\mathrm{TN}\right)-\left(\mathrm{FP}*\mathrm{FN}\right)}{\sqrt{\left(\mathrm{TP}+\mathrm{FN}\right)*\left(\mathrm{TP}+\mathrm{FP}\right)*\left(\mathrm{TN}+\mathrm{FN}\right)*\left(\mathrm{TN}+\mathrm{FP}\right)}}\;\mathrm{Range}\;\left[0,\;1\right]$$

(6) $$\mathrm{ACC}=\frac{\mathrm{TP}+\mathrm{TN}}{\mathrm{TP}+\mathrm{TN}+\mathrm{FP}+\mathrm{FN}}\;\mathrm{Range}\;\left[0,\;1\right]\;$$

(7) $$\mathrm{Jaccard}=\frac{\mathrm{TP}}{\mathrm{TP}+\mathrm{FP}+\mathrm{FN}}\;\mathrm{Range}\;\left[0,\;1\right]\;$$

(8) $$\mathrm{Precision}=\frac{\mathrm{TP}}{\mathrm{TP}+\mathrm{FP}}\;\mathrm{Range}\;\left[0,\;1\right]$$

(9) $$\mathrm{Recall}=\frac{\mathrm{TP}}{\mathrm{TP}+\mathrm{FN}}\;\mathrm{Range}\;\left[0,\;1\right]$$

(10) $$F1\;\mathrm{score}=\frac{2\times \mathrm{Precision}\;\times \mathrm{Recall}}{\mathrm{Precision}+\mathrm{Recall}}\;\mathrm{Range}\;\left[0,\;1\right]\;$$

(11) $$\mathrm{AUC}=\mathrm{Area}\;\mathrm{under}\;\mathrm{the}\;\mathrm{receiver}\;\mathrm{operating}\;\mathrm{characteristic}\;\mathrm{curve}\;\mathrm{Range}\;\left[0,\;1\right]$$

In Equations (1) and (2), N, $y_{i}$, $\hat{y}$, and $\bar{y}$ represent the total number of observations, the actual value for the ith observation, the predicted value of y, and the average value of y, respectively. In Equations (3)–(9), the numbers of true negatives (TN), false negatives (FN), true positives (TP), and false positives (FP) are used as the inputs of metric.

R^2^ is a statistical measure that represents the portion of the variance in the dependent variable that is predictable from the independent variable(s). The value ranges between 0 and 1, where 1 indicates that the model perfectly predicts the dependent variable. RMSE is a measure of the average magnitude of the error between predicted values and actual values. It is computed by obtaining the square root of the mean squared error. R^2^ and RMSE are both commonly used to evaluate the performance of regression models. They can be used to evaluate the performance of a model that predicts the metabolism and excretion outcomes. High R^2^ and low RMSE values indicate that the model can accurately predict the metabolism and excretion outcomes, while low R^2^ and high RMSE values suggest that the model cannot accurately predict the metabolism and excretion outcomes.

SE is the ratio of true positive predictions among all actual positive cases. SP is the proportion of true negative predictions among all actual negative cases. MCC is a measure influenced by all values including TP, TN, FP, and FN; it is a measure of the balance between true positives and true negatives. ACC is the proportion of correct predictions among all predictions. It measures the overall performance of the model. The Jaccard score, which is an intersection of union with respect to the minority class, is another obvious method to cope with unbalanced data [13]. Precision is the proportion of true positive predictions among all positive predictions. It measures the ability of the model to avoid false positive predictions. Recall (also known as sensitivity) is the proportion of true positive predictions among all actual positive cases. It measures the ability of the model to identify all the positive cases. The F1 score is the harmonic means of precision and recall. It is a measure that considers both precision and recall and it can be a better metric than precision and recall alone. AUC is a metric of a model’s ability to differentiate between positive and negative classes. AUC ranges from 0 to 1, where a value of 1 indicates that the model can perfectly distinguish between positive and negative cases, while a value of 0.5 indicates that the model is not able to distinguish between positive and negative cases. In AI-based drug metabolism and excretion prediction, these metrics are commonly used as evaluation metrics for classification problems.

There is no single metric that is superior in all cases. When evaluating and comparing the performance of different AI-based models for drug metabolism and excretion prediction, it is important to use appropriate evaluation metrics, use multiple evaluation metrics, make sure when comparing the model that all of the metrics are the same, consider the variability and uncertainty of the model’s performance, and consider the real-world implications of the model performance.

## 3. Drug Metabolism Prediction

**Figure 2:**
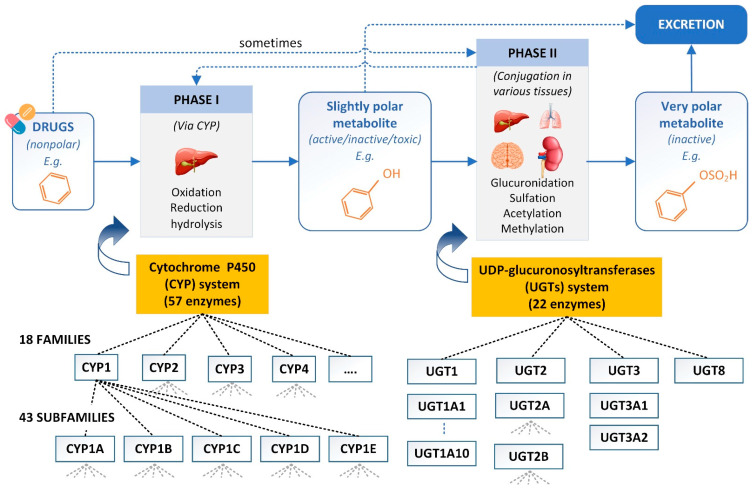
Phase I and phase II in drug metabolism.

Drug metabolism refers to the biochemical processes by which the body modifies and eliminates drugs and other foreign substances. The body metabolizes drugs to either activate or inactivate them, and this can influence their therapeutic effects and potential for toxicity. The process of drug metabolism reactions can be classified into two main phases based on their chemical nature [3,6] (Figure 2). Phase I metabolism typically involves the oxidation, reduction, or hydrolysis of the drug, which can produce metabolites that are either inactive, active, or toxic. This process is primarily carried out by a family of enzymes called cytochrome P450 (CYP) enzymes. Phase II metabolism involves the conjugation of the modified drug with another molecule, such as glucuronic acid, sulfate, or amino acids. This process increases the water solubility of the drug and makes it easier to excrete. Phase II reactions are typically carried out by a variety of enzymes, including UDP-glucuronosyltransferases (UGTs), sulfotransferases, and glutathione S-transferases (GSTs). It is crucial to keep in mind that these reactions do not have to happen in order; they could even occur in reverse, in phase II, then in phase I, or as a single reaction [14]. The rate and extent of drug metabolism can vary greatly depending on the specific drug and individual factors such as genetics, sex, age, and disease status. Some drugs may be metabolized very quickly, while others may be metabolized very slowly, leading to the accumulation of potentially toxic levels of the drug in the body.

The human CYP family has 57 isozymes [15]. CYPs are the primary enzymes involved in the metabolism of drugs, accounting for approximately 75% of overall metabolism, with about 95% of this activity being attributed to five isozymes, including 1A2, 2C9, 2C19, 2D6, and 3A4 [16]. The CYP-mediated metabolism of a novel chemical entity is of great importance during drug development because it has the potential to significantly impact the compound’s initial bioavailability, desired activity, and safety profile [17]. Quantitatively, UGTs accounted for 14% of the total metabolites collected, second only to the occurrence of CYP-catalyzed reactions [18]. Therefore, any study that leads to further insight into the mechanical aspects of metabolism will significantly support drug candidates’ rational design.

The majority of drug-related metabolism takes place in the liver, as the enzymes that facilitate the reactions are concentrated there. Some drugs can be inhibitors or inducers of metabolic enzymes. If one drug is an inhibitor of the metabolism of another drug, when the two drugs are taken together in the body, the exposure of the other drug may be higher than expected, leading to potential safety problems. If one drug is an inducer of an enzyme that metabolizes another drug, when the two drugs are used concurrently, the effect of the other drug may be lower than expected, leading to adverse potential pharmacological effects in the body. This phenomenon is commonly known as a drug–drug interaction [19]. Metabolism can also create metabolites that are good for medicine and toxic metabolites [18]. Therefore, enzymatic metabolism studies are used to resolve metabolic stability, quantify, and identify main metabolites, identify metabolic pathways, and assess the possibility of drug–drug interactions throughout the preclinical stage and drug discovery [20].

In silico, AI applications in the field of metabolic prediction fall into three major categories: (1) the sites of metabolism (SOMs) prediction, (2) metabolite structures prediction, and (3) metabolic pharmacokinetics prediction [10,21]. We provide recent developments of AI models in each category in the sections that follow.

### 3.1. SOMs and Metabolite Structure Predictions

The prediction of SOMs is critical for a xenobiotic since it gives critical information for the derivation of potential metabolites [22]. Chemists can usually predict the structure of a metabolite by knowing the atom position in the molecule where the metabolizing reaction is most likely to occur [23]. In silico approaches for predicting the SOMs and metabolite structures in CYP-mediated processes are commonly used as a starting point for metabolic pathway research, which can also help with drug/lead optimization. Much software to predict SOMs for phases I and II has been developed, such as FAME [24], FAME 2 [25], FAME 3 [26], GLORY [23], GLORYx [27], BioTransformer [28], CypReact [13], CyProduct [29], and PreMetabo [30], summarized in Table 1.

CypReact software uses machine learning (ML) to predict when a small chemical will react with any of the nine critical CYP isozymes. It employs a random forest (RF) model for each of the seven isozymes (1A2, 2A6, 2B6, 2C8, 2C19, 2E1, 3A4) and ensemble models (RF, support vector machine (SVM), logistic regression, and decision tree) for the remaining two isozymes (2C9, 2D6). Each model predicts substrate specificity based on a set of structural features and physicochemical properties of a molecule. Authors used 679 compounds from XenoSite [31] and manually gathered 1053 unreacted compounds to enhance the quality and predictability of the dataset, including known medicines, pesticides, dietary components, pollutants, endogenous metabolites, and a range of other substances. CypReact’s classifiers produce extremely high performance, with AUC scores between 83 and 92%. Additionally, CypReact is statistically superior to the baseline, according to a simple paired *t* test, with *p* values < [4.17E–6, 2.60E–4, 2.36E–5, 1.46E–4, 4.41E–5, 5.01E–6, 3.23E–5, 6.44E–6, 3.25E–6] for the nine CYPs. With *p* values < [4.90E–6, 5.25E–6, 1.54E–7], CypReact is statistically superior to SMARTCyp [32] for all three of the studied isoforms.

Developed by the same author team, CyProduct is an in silico metabolism predictor to accurately predict the byproducts of human CYP metabolism. It consists of three tools: (1) CypReact predicts whether the query compound reacts with a specific CYP enzyme; (2) CypBoM Predictor predicts the reaction’s “bond site”; and (3) MetaboGen produces metabolic byproducts based on the bond-site prediction of CypBoM. It predicted the metabolic biotransformation products of the nine most essential human CYP enzymes: 1A2, 2A6, 2B6, 2C8, 2C9, 2C19, 2D6, 2E1, and 3A4. CypBoM makes use of a novel notion called “bond of metabolism” (BoM), which complements the classic “site of metabolism” by identifying the collection of chemical bonds that are changed or created during a metabolic reaction. A BoM dataset for 1845 CYP-mediated phase I reactions was created, and it was used to train the CypBoM predictor to anticipate the reactive bond position on substrate molecules. The cross-validated Jaccard score generated by CypBoM Predictor ranged between 0.380 and 0.452 for reactive bond prediction for the nine CYP enzymes. CypBoM Predictor’s Jaccard score is 0.13 better than that of FAME 2 and 0.12 better than that of FAME 3 in terms of SOMs on the 86 compounds. Moreover, CyProduct surpassed the other software tools, including ADMET Predictor, GLORY, and BioTransformer, in predicting metabolites by an average of 200% across variants of a testing dataset of 68 CYP substrates and 30 non-reactants concerning Jaccard scores. More specifically, for the BioTransformer dataset, the performance of CyProduct is about 30% better than that of BioTransformer and ADMET Predictor. Among the above software packages, CyProduct software is the most recently published and has a relatively detailed and clear performance comparison with others.

**Table 1:** Public metabolism prediction tools.

Name	Metabolism Prediction	Methods	Website *	Ref.
CyProduct (CypReact, CypBoM, MetaboGen)	Reactant, BoM for CYP, metabolite structure	ML	https://bitbucket.org/wishartlab/cyproduct/src/master/	[29]
GLORYx	Metabolite structure	ML	https://nerdd.univie.ac.at/gloryx/	[27]
FAME 3	Phase 1 and 2 SOMs for CYP	ML	https://nerdd.univie.ac.at/fame3/	[26]
BioTransformer 3.0	Metabolic transformation	rule-based/knowledge-baseb, ML	http://biotransformer.ca/	[28]
PreMetabo	Phase 1 and 2 SOMs for CYP, UGT, and SULT	Arrhenius equation and EaMEAD model	https://premetabo.bmdrc.kr/	[30]
SMARTCyp 3.0	SOMs for CYP	rule-based	http://smartcyp.sund.ku.d/	[33]
HelixADMET	CYP inhibitors and substrates	GNN	https://paddlehelix.baidu.com/app/drug/admet/train	[34]
Interpretable-ADMET	CYP inhibitors and substrates	GAT, GCNN	http://cadd.pharmacy.nankai.edu.cn/interpretableadmet/	[35]
FP-ADMET	CYP inhibitors and substrates	RF	https://gitlab.com/vishsoft/fpadmet	[36]
ADMETlab 2.0	CYP inhibitors and substrates	GCNN	https://admetmesh.scbdd.com/	[37]
AdmetSAR 2.0	CYP inhibitors and substrates	RF, k-NN, SVM	http://lmmd.ecust.edu.cn/admetsar2/	[38]
SwissADME	CYP inhibitors	MLR, RNN, SVM	http://www.swissadme.ch/	[39]
ICDrug ADMET	CYP inhibitors and substrates	RF	www.icdrug.com/ICDrug/ADMET	[40]
Virtual Rat	CYP inhibitors	RF	https://virtualrat.cmdm.tw/	[9]
DL-CYP	CYP inhibitors	DNN	http://www.pkumdl.cn/deepcyp/home.php	[41]
CYPstrate	CYP substrates	RF, SVM	https://nerdd.univie.ac.at/cypstrate/	[42]
CYPlebrity	CYP inhibitors	RF	https://nerdd.univie.ac.at/cyplebrity/	[43]
SuperCYPsPred	CYP inhibitors	RF	http://insilico-cyp.charite.de/SuperCYPsPred/	[44]

* Websites were accessed on 15 October 2022. Abbreviations: k-NN: k-nearest neighbor, MLR: multiple linear regression, RNN: recurrent neural network, DNN: deep neural network, GCNN: graph convolutional network, GAT: graph attention network.

FAME, FAME 2, GLORY, FAME 3, and GLORYx are extensive series of legacy metabolic prediction software, respectively. The latest version, GLORYx, has extended the approach from GLORY, which combines SOMs prediction with a collection of reaction rules to predict phase I and II metabolism. Researchers used the SOMs probabilities prediction by the FAME 3 ML model on the SOMs dataset containing 1748 parent molecules from MetXBioDB [28] and the DrugBank database to achieve the predicted metabolites. FAME 3 uses extremely randomized tree classifiers and circular descriptors, including 15 basic 2D CDK descriptors and circular atom-type fingerprints. On a curated test dataset collecting phase I and phase II metabolites, GLORYx achieved an AUC of 0.79 and a recall of 77%. This performance was better than that of the GyGMa tool [45] on the same dataset but not better when regarding only phase II metabolite prediction. Furthermore, the authors note that it is difficult to give a firm definition of the area of application of GLORYx due to the scarcity of available high-quality data on small-molecule metabolism.

### 3.2. CYP Inhibitor and Substrate Prediction

As mentioned above, the CYP enzyme family plays a crucial role in drug metabolism. CYP inhibitors can affect the metabolism of drugs by reducing the activity of the CYP enzymes involved in their metabolism, leading to changes in the pharmacokinetics and pharmacodynamics of the drugs. On the other hand, the rate and extent of CYP substrate metabolism can be influenced by the presence of CYP inhibitors or other factors. Predicting the potential of a drug to be a CYP inhibitor or substrate is a complex process that involves many factors and variables, including the individual’s genetic makeup, age, and overall health. As such, predictions may not always be accurate and may need to be confirmed through further testing and analysis.

Moreover, especially in recent years, there have been a lot of studies focusing on the prediction of specific metabolic CYP isoforms with remarkably good performance. Many prominent studies have focused on predicting the 5 major CYP inhibitors (1A2, 2C19, 2C9, 2D6, and 3A4), DeepCYP [41], SuperCYPsPred [44], CYPlebrity [43], iCYP-MFE [46], VirtualRat [9], and others [47,48]. Some studies focused on CYP substrate prediction, such as [33,47,49]. Some studies have focused on predicting only one CYP, such as CYP1B1 [50], CYP1A2 [51], CYP2C8 [52], CYP2C9 [53,54], and CYP3A4 [55,56]. Their performances are summarized in detail in Table 2.

**Table 2:** Summary of AI methods to predict CYP subtypes from 2019 to 2022.

CYP Subtypes	Methods	Data Sources	Dataset Size (Compounds)	Best Performance	Ref.
1A2, 2C19, 2D6, 2C9 and 3A4 inhibitors	RF	PubChem, SuperCYP	18,313	ACC = 0.97, AUC = 0.98	[44]
1A2, 2C9, 2C19, 2D6 and 3A4 inhibitors	RF	ChEMBL, PubChem, ADME	134,844	AUC = 0.92, ACC = 0.83	[43]
1A2, 2D6, 2C9, 2C8, 2C19, and 3A4 inhibitors	RF	[52,57]	17,652	ACC = 0.868, AUC = 0.741	[36]
CYPs 1A2, 2C9, 2C19, 2D6 and 3A4 inhibitors	RF, SVM, k-NN	PubChem	65,467	AUC =0.93	[46]
1A2, 2D6, 2C9, 2C19, and 3A4 inhibitors 2D6, 2C9, and 3A4 substrate	RF, SVM, k-NN	[57]	77,490 2018	ACC = 0.855, AUC = 0.84	[38]
1A2, 2D6, 2C9, 2C19, and 3A4 inhibitors	DT	[58,59]	64,129	ACC = 0.93, Recall = 0.924	[9]
1A1, 1A2, 2A6, 2B6, 2C8, 2C9, 2C19, 2D6, 2E1, and 3A4 substrates	Improved Bayesian method	SuperCYP [60], PubChem, DrugBank, CYP450 Engineering Database [61,62], Meta-CYP	7114	AUC = 0.92, ACC = 0.90	[49]
1A2, 2C19, 2C9, 2D6, and 3A4 inhibitors 1A2, 2C19, 2C9, 2D6, and 3A4 substrates	MGAF	ChEMBL, PubChem, OCHEM, literature	62,771 (inhibitors) 3291 (substrates)	ACC = 0.886, AUC = 0.948	[37]
1A2, 2C19, 2C9, 2D6, and 3A4 inhibitors2C9, 2D6, and 3A4 substrates	GCNN, GAT	ChEMBL, PubChem, DrugBank, literature	63,921 (inhibitors)2053 (substrates)	ACC = 0.85, AUC = 0.93	[35]
1A2, 2C19, 2C9, 2D6, and 3A4 inhibitors 1A2, 2C19, 2C9, 2D6, and 3A4 substrates	GNN	PubChem, CypReact [13], SuperCYP [44]	64,801 (inhibitors) 9233 (substrates)	AUC = 0.967	[34]
1A2, 2D6, 2C9, and 2C19 inhibitors	RF, GBDT, XGB, DNN, CNN	[41]	53,179	ACC = 0.974, AUC = 0.991	[63]
1A2, 2C9, 2C19, 2D6 and 3A4 inhibitor	MT-DNN	PubChem	153,484	AUC = 0.937, ACC = 0.895	[48]
2C8 inhibitors	RF, SVM, k-NN, LR, ANN	PubChem and literature [64,65]	514	AUC = 0.90, ACC = 0.89	[52]
2C9 inhibitors	RF, SVM	ChEMBL	8141	ACC = 0. 843, MCC = 0.695	[54]
2C9 inhibitors	BT, multilayer feedforward of resilient backpropagation network	PubChem	>35,000	AUC = 0.85	[53]
3A4 inhibitors	GCNN combined with the MT-DNN	ChEMBL and [66]	3774	R^2^ = 0.692	[67]
			89,619	R^2^ = 0.414	
3A4 inhibitors	SVM, XGB, and RF	In-house, public	30,768	ACC = 0.927, sensitivity = 0.788	[56]
		In-house	26,138	ACC = 0.90, AUC = 0.908	
1B1 inhibitors	RF, SVM, ANN	ChEMBL, Pubchem, and [68,69,70]	714	MCC = 0.95	[50]
1A1 inhibitors			658	MCC = 0.96	
1A2 inhibitors	CNN	PubChem	21,721	ACC = 0.722, AUC = 0.819	[51]

Abbreviations: ANN: artificial neural networks, CNN: convolutional neural networks, GNN: graph neural network, MT-: multitask-, DT: decision trees, GBDT: gradient boosting decision trees, LR: linear regression, XGB: extreme gradient boosting, BT: boost tree, MGAF: a multi-task graph attention framework.

SuperCYPsPred [44] is a free, friendly, and publicly accessible web application that employs well-established ML techniques to predict five key CYP inhibitors, including 1A2, 2C19, 2C9, 2D6, and 3A4. The model was constructed using RF and various types of data sample methods on a dataset of 1170 pharmaceuticals with Morgan and MACCS circular fingerprints from their in-house SuperCYP and PubChem databases. SuperCYPsPred is extremely accurate, with an average cross-validation ACC of 93% and an average external validation ACC of 88.2%. SuperCYPsPred is among the most effective free tools for CYP prediction, making it a good preclinical drug discovery and development screening tool.

In addition, some large recently developed ADMET (absorption, distribution, metabolism, excretion, and toxicity) prediction tools also integrate CYP prediction with high efficiency, such as HelixADMET [34], Interpretable-ADMET [35], FP-ADMET [36], ADMETLab 2.0 [37], and admetSAR 2.0 [38]. Many other prominent tools are summarized in Table 1. We also summarize in detail in Table 2 the AI models developed since 2019 that focus on predicting CYP subtypes. It is important to note that while these methods can provide useful information, they are not always predictive of the effects seen in vivo, and further in vivo studies are needed to fully understand the impact of CYP inhibition and substrate interactions on drug metabolism and efficacy.

### 3.3. UGTs Prediction

Glucuronosyltransferases are responsible for the glucuronidation process, a primary and most important part of phase II metabolism [71]. The UGT enzyme [15] catalyzes the addition of a glucuronic acid moiety to xenobiotics, which is the primary method through which the human body eliminates the most frequently prescribed medications. It is also the primary route of chemical elimination for the majority of medications, dietary agents, poisons, and endogenous compounds from the diet, environmental sources, and pharmaceutical industries. However, compared with phase I reactions, phase II metabolism is much less noticeable, although it has an important impact on modulating pharmacological effects [72]. Predicting the potential of a drug to be metabolized by UGTs is an important aspect of drug development, as it can help to determine the risk of drug–drug interactions and potential adverse effects. Table 3 summarizes several studies predicting UGTs in recent years.

Mazzolari et al. built two models using molecular descriptors and RF algorithms to predict UGT-mediated metabolism [73]. The first model predicts whether a molecule is prone to conversion to glucuronide using 2192 molecules from the MetaQSAR database [74], achieving an AUC of 0.94 and an MCC of 0.76 in the internal evaluation and an AUC of 0.90 and an MCC of 0.70 in external evaluation using 120 additional xenobiotics. The second model differentiates between the two major forms of glucuronidation by determining whether conjugation takes place on an oxygen or nitrogen atom (O- or N-glucuronidation) using 661 O-glucuronidation and 114 N-glucuronidation substrates, with a recall value of 0.78. This result emphasizes the need to utilize well-curated datasets when developing new methodologies for predicting phase II metabolism and demonstrates a practical application of the MetaQSAR database.

The SOM prediction model was developed by Cai et al. [75] for four subtypes of UGT-mediated reactions, including AlOH, ArOH, COOH, and nitrogen, using DT, RF, and AdaBoost methods. They used 400 drugs metabolized by UGT from the *Handbook of Metabolic Pathways of Xenobiotics* [76] and two external test sets from previous studies [77,78,79]. Differently sized atom environment fingerprints were used to describe the SOMs. The best performance of their optimal models yielded an ACC of 86.7% on the test set and 79.8% on the external test sets. However, the use of small and undiversified datasets is one of their limitations, which hinders the full exploitation of the possibilities of ML methods and the performance is not appreciated.

PreMetabo [30] is an available web tool to predict phases I and II drug metabolism using knowledge-based prediction models. For phase I drug metabolism prediction, Hwang et al. used the EaMEAD model based on the Arrhenius equation for four CYP enzymes (1A2, 2C9, 2D6, 3A4) to predict the SOMs on the drug molecule. In phase II, they developed a consensus classification model using SVM to predict the UGT and SULT substrate. PreMetabo used 200 substrates of each CYP from Fujitsu ADME database for evaluation and comparison with the BioTransformer tool [28]. The predictability of the primary metabolite in the top-3 was determined to be from 72.5 to 84.5% for four CYPs in the SOMs prediction model. The PreMetabo recall value of all CYPs was higher than the BioTransformer recall value on the same dataset and it was judged to be more practical than BioTransformer. Besides, in phase II, they used UGT and SULT substrates from MDL metabolism and Fujitsu ADME databases with 1024 ECFP4 fingerprints and 881 PubChem fingerprints. The highest accuracy of their models was determined to be 93.9 and 80.7%, respectively, for internal validation. Moreover, PreMetabo achieved a UGT substrate prediction ACC of 81% on the external test dataset containing 11 FDA-approved drugs.

**Table 3:** Summary of AI methods to predict UGT property from 2019 to 2022.

Methods	Data Sources	Dataset Size (Compounds)	Performance	Ref.
RF	MetaQSAR	7962	MCC = 0.76, AUC = 0.94	[73]
DT, RF, AdaBoost	*Handbook of Metabolic Pathways of Xenobiotics* [76], KEGG [77,78,79]	586	ACC = 0.867, AUC = 0.928	[75]
LR, SVM	Fujitsu ADME database and MDL metabolism database	200	ACC = 0.81	[30]

## 4. Drug Excretion Prediction

Drug excretion is the process through which medications are excreted from the body, as metabolites or as original drugs [4]. Excretion is a complicated process involving many elimination routes. The kidneys are in charge of excreting the majority of chemicals that are water-soluble. Additionally, the biliary system can excrete medications that are not absorbed by the stomach tract. The number of drugs eliminated by the intestines, saliva, sweat, breast milk, and lungs is usually insignificant. On the other hand, certain volatile anesthetics are capable of being exhaled via the lungs. Additionally, even minute amounts of the drug in a nursing woman’s breast milk can have an effect on her nursing infant. During development, drug excretion properties contribute to the validation of toxicity studies, aid in assessing safety before the first dose in humans, provide dosimetry data in humans for the clinical, and indicate the possibility of drug–drug interactions. The main pharmacokinetic parameters for drug excretion include clearance and half-life (t_1/2_). In the next section, we present recent advances in predictive research clearance and t_1/2_ properties using AI.

### 4.1. Clearance Prediction

The volume of plasma cleared of a drug over a given time period is referred to as drug clearance [80]. As a result, the unit of measurement for drug clearance is volume/time. Another equation can be used to compute the discharge. Clearance is computed by dividing the rate of elimination of a drug from plasma (mg/min) by its concentration in plasma (mg/mL). The entire ability of the body to eliminate the medication from plasma is comprised of renal clearance, hepatic clearance, and clearance from all other tissues. Clearance may be affected by body weight and surface area, cardiac output, renal function, liver function, plasma protein binding, concomitant medications, and changed expression presence of drug-metabolizing enzymes [81]. Clearance is a critical pharmacokinetic parameter to consider in both drug discovery and clinical practice because clearance is a factor of all other relevant pharmacokinetic parameters, including half-life, oral bioavailability, and effective dose [82]. Many recent studies predicting clearance property in silico are summarized in Table 4.

Recently, software has been developed to predict ADMET properties named FP-ADMET, which integrates clearance prediction including human renal clearance, intrinsic clearance, metabolic intrinsic clearance, and human liver microsomal clearance [36]. Researchers used a fingerprint-based RF algorithm for the four-clearance prediction models. The data used to evaluate the four models were 636 compounds, 244 compounds, 5278 compounds, and 5348 compounds, respectively, which were collected from many previous studies [66,83,84,85]. The human renal clearance prediction model of FP-ADMET gave better predictive results than the study of Chen et al. [85] on the same dataset and algorithm with R^2^ of 0.27 and RMSE of 0.53 (compared to R^2^ = 0.2 and RMSE = 1.8). However, the intrinsic clearance prediction model of FP-ADMET showed no better predictive results than the study of Hsiao et al. [84] on the same dataset with R^2^ of 0.29 (compared to R^2^ = 0.96). The accuracy of the metabolic intrinsic clearance model by FP-ADMET was 74%, higher than the accuracy of the RF model used by Esaki et al. [83] on the same dataset (72.3%), but the radial SVM model used by Esaki et al. had a higher ACC of 77.1%. The performance of the human liver microsomal clearance model by FP-ADMET was no better than that of the MT-DNN model used by Wenzel et al. [66] on the same dataset, with an R^2^ of 0.56 (compared to R^2^ = 0.624). The RF model developed by Wang et al. for the clearance prediction also achieved higher performance than other models such as SVM, GBM, and XGB on 1352 compounds, with an R^2^ of 0.875 and an RMSE of 0.103 [86]. Furthermore, Kosugi and Hosea once again proved that the total plasma clearance prediction model using the RF algorithm is more efficient than many other algorithms, such as radial basis function fitting (RBF), partial least squares (PLS), random forest regression (RFR), Gaussian process models (GP) with two-dimensional search for parameters (GP2DS), fixed hyperparameters (GPFixed), hyperparameters obtained by forward variable selection (GPFVS), rescaled procedure (GPRFVS), and by conjugate gradient optimization (GPOPT) on the same dataset of 1114 compounds with an RMSE of 0.4 using five-fold cross-validation [87]. The best performing human renal clearance prediction model developed by Watanabe et al. is also a model using the RF algorithm when compared with other algorithms such as SVM, PLS, and ANN on 401 compounds, with an R^2^ of 0.92 and an RMSE of 0.12 [88]. With many years of experience in ADMET prediction and a large internal dataset of 73,620 compounds, AstraZeneca built a clearance prediction model using the SVM algorithm, with good results with an RMSE of 0.377 [89].

In addition to ML algorithms, recently deep learning (DL) algorithms have also been exploited and built predictive models of clearance with remarkable efficiency. Mamada et al. successfully combined conventional ML using molecular descriptors with DeepSnap-DL to build a new clearance prediction model [90]. They used rat clearance data containing 1545 in-house compounds to evaluate the prediction performance. With an AUC and an ACC of 94.3 and 87.4%, respectively, their ensemble model did better than conventional ML (AUC = 88.3% and ACC = 82.5%) or DeepSnap-DL (AUC = 90.5% and ACC = 83.2%). Sohlenius-Sternbeck et al. developed an intrinsic clearance prediction model using an ANN algorithm and 4794 compounds from Medivir in-house dataset [91]. This model was a significant improvement over ADMET PredictorTM from Simulations Plus, with R^2^ of 0.717 (compared to R^2^ = 0.53). Using the same dataset of 5384 compounds, the combined model of GCNN and MT-DNN model of Liu et al. [67] and the MT-DNN model of Wenzel et al. [66] achieved approximately equal accuracy in clearance prediction (R^2^ = 0.62). Recently, DL technical was also exploited in ADMETLab 2.0 using MGAF to predict clearance on 831 compounds, achieved an R^2^ of 0.629 [14].

**Table 4:** Summary of AI methods to predict clearance property from 2019 to 2022.

Methods	Data Sources	Dataset Size (Compounds)	Performance	Ref.
RF	Human renal clearance [85]	636	R^2^ = 0.27, RMSE = 0.53	[36]
	Intrinsic clearance [84]	244	R^2^ = 0.29, RMSE = 1.02	
	Metabolic intrinsic clearance [83]	5278	ACC = 0.74, AUC = 0.84	
	Human liver microsomal clearance [66]	5348	R^2^ = 0.56, RMSE = 1.05	
RF, SVM, GBM, XGB	[92]	1352	R^2^ = 0.875, RMSE = 0.103	[86]
RFR, RBF, PLS, GP2DS, GPFixed, GPFVS, GPRFVS, GPOPT	Takeda Pharmaceutical Company (Fujisawa, Japan)	1114	R^2^ = 0.61, RMSE = 0.31	[87]
SVM	AstraZeneca in-house data	73,620	R^2^ = 0.356, RMSE = 0.377	[89]
RF, NB, SVM, CT, k-NN, MLR, ANN	FDA drugs and [93,94,95,96]	636	R^2^ = 0.94, RMSE = 0.11	[85]
RF, AdaBoost, Radial SVM, Linear SVM	ChEMBL v.23, KEGG DRUG [97]	56,065	ACC = 0.77, Kappa = 0.588	[83]
RF, SVM, PLS, ANN	ChEMBL and Varma et al. [98]	401	R^2^= 0.92, RMSE = 0.12	[88]
Combination conventional ML and DeepSnap-DL	in-house	1545	AUC = 0.943, ACC = 0.874	[90]
ANN	Medivir in-house	4794	R^2^ = 0.717, RMSE = 0.327	[91]
GCNN	ChEMBL, PubChem, OCHEM, literature	831	R^2^ = 0.692	[37]
a molecular GCNN combined with the MT-DNN	[66]	5348	R^2^ = 0.62	[67]
	Amgen’s internal datasets	86,470	R^2^ = 0.445	
MT-DNN	ChEMBL v.23	5384	R^2^ = 0.624	[66]
MT-CNN	AstraZeneca	139,907	R^2^ = 0.59, RMSE = 0.35	[99]

Abbreviations: NB: Naïve Bayes, CT: classification tree, SVR: support vector regression.

### 4.2. Half-Life Prediction

The excretion half-life of the drug is the length of time needed for the amount of the active component in the drug to decrease by half of its starting dose in the body [100]. This is dependent on how the substance is metabolized and eliminated by the body. It can last anywhere from a few hours to several days or even weeks. Understanding the concept of half-life makes it possible to calculate the steady-state concentrations and excretion rates for any given drug. A more frequent dosage may be necessary to maintain the proper level of exposure and prevent unnecessary peak concentrations if a drug’s half-life is too short [101]. As a result, it could be more challenging to achieve the best efficacy, safety, and patient compliance. A drug’s extremely lengthy half-life may increase the amount of time needed for subsequent accumulation and elimination. This can complicate the management of adverse events and the design of efficient clinical trials. An accurate estimate of the time needed for medicine or substance to be excreted from the body is difficult to come by. Some of the models developed since 2019 are summarized in Table 5.

Interpretable-ADMET, a new ADMET predictor, uses GCNN and GAT algorithms to predict 59 ADMET properties, including half-life [35]. The GAT model gave a slightly better half-life prediction result than the GCNN model, with an ACC of 77.6 and 77.3%, respectively, on 665 compounds. In ADMETLab 2.0, a multi-task graph attention framework was used to build the ADMET prediction models, including half-life prediction model [37]. The half-life prediction model was evaluated on 1219 compounds and had a predictive ACC of 74% and an AUC of 82%. Furthermore, the software that has been developed for predicting ADMET properties named FP-ADMET also has integrated half-life prediction [36]. Researchers used a fingerprint-based RF algorithm and 2127 compounds from MetStabOn [102] to predict half-life. When using the same dataset and RF algorithm, FP-ADMET predicts half-life more accurately than MetStabOn, with an ACC of 76 and 72.6%, respectively. In another study, Wang et al. built a predictive model of the half-life and three other properties using ML methods, including RF, gradient boosting machines (GBM), SVM, and XGB [86]. They used a dataset of 1352 compounds from Lombardo et al. [92] and 162 critical variables, including 2D molecular, 3D molecular, and fingerprint descriptors. Assessed by 10-fold cross-validation, the RF model produced more accurate prediction than other models, with an R^2^ of 0.832 and an RMSE of 0.154. The actual half-life of the same drug can differ considerably between individuals due to a variety of patient- and drug-specific characteristics. Therefore, in silico studies predicting drug half-life are also very limited. There is hardly a single recent half-life predictive study. Most half-life prediction models are mainly integrated into large ADMET prediction programs or researched with many other properties.

The excretion of a drug is a complicated process involving many elimination pathways, including biliary excretion, renal excretion, and others, each of which includes many different processes [22]. Till now, in silico excretion predictors have been difficult to develop due to the complex drug excretion processes.

**Table 5:** Summary of AI methods to predict half-life property from 2019 to 2022.

Methods	Data Sources	Dataset Size (Compounds)	Performance	Ref.
RF	[102]	2127	ACC = 0.76, AUC = 0.88	[36]
SVM, RF, GBM, XGB	[92]	1352	R^2^ = 0.832, RMSE = 0.154	[86]
MGAF	ChEMBL, PubChem, OCHEM, literature	1219	AUC = 0.822, ACC = 0.744	[37]
GCNN, GAT	ChEMBL, PubChem, DrugBank, literature	665	ACC = 0.773, AUC = 0.766	[35]

Although researchers have proposed many AI-based models to predict drug metabolism and elimination, evaluating and comparing them on an objective basis can be quite challenging. A lack of consensus datasets and evaluation metrics can be a major limitation in comparing predictive models in the field of biology and biomedicine. In Table 2, Table 3, Table 4 and Table 5, we summarize recently developed AI-based methods for drug metabolism and elimination prediction. We only provide comparative information when the authors used the same dataset and metrics to evaluate their models in the content.

## 5. Data Sources for Research Community

The selection of an appropriate database is a critical step in the development of accurate and reliable AI-based predictive models for metabolism and excretion. Careful consideration should be given to the quality, completeness, and relevance of the data in order to ensure the best possible results. Some commonly used databases to predict drug metabolism and excretion are briefly described as follows:

- HMDB 5.0 (https://hmdb.ca/ accessed on 22 January 2023): An extensive database of small molecule metabolites discovered in the human body, including information on their chemical and physical properties, metabolic pathways, and clinical biomarkers. Information on more than 220,000 metabolites and 8500 protein sequences can be found in HMDB. [103].
- METLIN (https://metlin.scripps.edu/ accessed on 22 January 2023): a metabolite database that contains information on more than 960,000 compounds [104]. It includes information on the chemical structure, molecular formula, and biological activities of metabolites. METLIN offers MS/MS data on various collision energy values in both positive and negative ionization modes. Additionally, it makes use of the elemental makeup, precise mass measurements, and the known structure of the metabolite to estimate the fragmented structure. The metabolomics-specific mobile interface METLIN Mobile allows you to see metabolite information from any cellular device.
- MetaCyc (https://metacyc.org/ accessed on 23 January 2023): A curated database of metabolic pathways and enzymes for a range of organisms. It includes information on 3085 pathways, 18,785 metabolites, and 18,391 reactions involved in metabolite biotransformation and can be used to construct metabolic models for specific organisms.
- MetaQSAR: A database for metabolites including information on the relationship between the chemical structure of a metabolite, its biological activity, the physicochemical properties of chemicals, as well as their predicted metabolic pathways and associated enzymes. It is a plug-in embedded in the VEGA ZZ programs (http://www.vegazz.net/ accessed on 23 January 2023) and contains 1890 substrates [74].
- MetXBioDB (https://bitbucket.org/djoumbou/biotransformerjar/src/master/ accessed on 23 January 2023): A database of metabolic pathways and enzymes for a range of organisms, including bacteria, archaea, and eukaryotes. MetXBioDB contains data on more than 2000 biotransformation including information on the structure and function of enzymes, as well as the reactions and pathways involved in metabolite biotransformation [28].
- Metabolights (https://www.ebi.ac.uk/metabolights/ accessed on 24 January 2023): A database of metabolomic data, which includes information on metabolites, metabolic pathways, and metabolic networks of more than 27,500 compounds. Metabolights also includes tools for data analysis and visualization, as well as resources for sharing and reusing metabolomic data [105].
- KEGG Pathway (https://www.genome.jp/kegg/pathway.html accessed on 24 January 2023): A database of metabolic pathways, including maps and diagrams of metabolic networks, as well as information on enzymes and metabolites. It includes information on more than 17,000 metabolic pathways and over 22,000 enzymes [106].
- HumanCyc (https://humancyc.org/ accessed on 24 January 2023): A curated database of metabolic pathways, enzymes for human metabolism, and the human genome. HumanCyc includes information on the reactions and pathways involved in metabolite biotransformation, as well as the enzymes and genes involved in these processes. Information on 28,783 genes, their products, and the metabolic processes and pathways they catalyze is contained in the pathway/genome database that was created as a consequence [107].
- BiGG (http://bigg.ucsd.edu/ accessed on 24 January 2023): In order to simulate systems biology and predict metabolic flux balance, the BiGG database reconstructs human metabolism metabolically. The 1496 ORFs, 2004 protein complexes, 2766 metabolites, and 3311 metabolic and transport processes are all included in this thorough literature-based genome-scale metabolic reconstruction. It was put together from building 35 of the human genome [108].
- DrugBank (http://www.drugbank.ca/ accessed on 24 January 2023): A comprehensive database of drug and drug target information including information on drug metabolism and pharmacokinetics, as well as the enzymes involved in drug biotransformation. It contains information on more than 500,000 drugs and their associated targets, pathways, and metabolic pathways [109].
- ChEMBL (www.ebi.ac.uk/chembl/ accessed on 24 January 2023): A database of bioactive molecules, including drugs and drug candidates, with information on their activities, targets, and metabolic pathways. It contains data on more than 2.3 million compounds and their associated activities and targets [110].
- ChemSpider (http://www.chemspider.com/ accessed on 24 January 2023): A chemical structure database that includes information on more than 115 million compounds including information on chemical structures, properties, and associated metadata, such as chemical identifiers and references [111].
- PubChem (https://pubchem.ncbi.nlm.nih.gov/ accessed on 25 January 2023): A public database of chemical structures and their associated biological activities including information on more than 114 million compounds, as well as tools for data analysis and visualization [112].
- ZINC20 (https://zinc20.docking.org/ accessed on 25 January 2023): A database of commercially available compounds for drug discovery including information on more than 750 million purchasable compounds, as well as tools for searching and filtering compounds based on various criteria, such as molecular weight, bioavailability, and toxicity [113].
- OCHEM (https://ochem.eu/home/show.do accessed on 25 January 2023): A platform for the development and validation of predictive models for chemical and biological data. OCHEM includes tools for data preprocessing, feature selection, and model training, as well as a library of pre-trained models. OCHEM contains more than 3.7 million records for 689 properties [114].
- Therapeutics Data Commons (TDC) (https://tdcommons.ai/ accessed on 25 January 2023): A database of clinical trial data for FDA-approved drugs including information on drug pharmacokinetics, pharmacodynamics, and adverse events, as well as data on drug metabolism and excretion. TDC contains data on more than 4.2 million compounds, 34,000 genes, and approximately 2 million reactions [115].
- openFDA (https://open.fda.gov/ accessed on 25 January 2023): A database of FDA-approved drugs, including information on drug labeling, adverse events, and clinical trial data. OpenFDA includes tools for data analysis and visualization, as well as an API for accessing FDA data [116].

These databases and datasets are publicly available, so they can be valuable resources for researchers working on metabolism and excretion prediction, pharmacokinetic property prediction, drug discovery, and related areas.

## 6. Challenges in Drug Metabolism and Excretion Prediction Based on AI

AI-based drug metabolism and excretion prediction presents the possibility of revolutionizing drug R&D, but there are still several issues that must be resolved to raise the accuracy and reliability of predictive models.

Metabolism and excretion are complicated biological processes involving multiple enzymes, transporters, biochemical pathways, multiple organs, and other molecular components. The interaction of these various components can be difficult to accurately model, resulting in inaccurate predictions. Predicting a drug’s metabolism necessitates an understanding of the relevant pathways and enzymes. Human metabolism and excretion are very diverse and are influenced by a variety of factors, including age, gender, heredity, illness status, interacting drugs, dose, and the route of administration [117]. Accurately predicting properties for all individuals is difficult. Genetic variations in these enzymes can lead to differences in drug metabolism and elimination between individuals, which can make it difficult to predict how a drug will be metabolized and excreted in different populations. Many drugs have complex metabolic processes that involve multiple enzymes and pathways. These pathways are frequently interrelated, and predicting the activity of a single enzyme or pathway may not be sufficient to predict overall metabolism and excretion of a drug. Because of the existence of metabolic intermediates that would allow for intramolecular rearrangement, it is uncertain that the basic mechanism and regulations of drug metabolism can be characterized only based on the drug structures [15]. Nonlinearities in the input rate (for example, formation) and the output rate (for example, elimination) can all have an impact on the distribution of metabolites [118]. The mechanisms underlying drug metabolism and excretion are not fully understood, particularly for some drug classes, which can hinder the development of accurate prediction models.

The lack of high-quality data is a significant challenge in developing accurate metabolism and excretion prediction models as well as AI-based drug discovery models in general [119]. Poor or incomplete data as well as erroneous data collection and analysis methods will lead to inaccurate predictions as the quality of the output is controlled by the quality of the input. AI algorithms require large amounts of comprehensive and high-quality experimental data to train and validate prediction models. However, generating comprehensive experimental data that accurately reflects these complex processes can be difficult. Experiments to measure drug metabolism and excretion can be time-consuming and costly, requiring specialized equipment and expertise [5]. As a result, there may be insufficient resources to generate the required data for all drugs of interest. In experimental studies, a lack of standardization and quality control measures can lead to variability and errors in the data, affecting the accuracy of AI models. The chemical diversity of compounds can make it challenging to develop universal prediction models that can accurately predict metabolism and excretion properties for a broad range of compounds. Data on drug metabolism and excretion can be collected from a variety of sources, including in vitro and in vivo experiments, clinical trials, and literature sources. These data sources may use different experimental methods, formats, and standards, and may have varying levels of quality, which can make it challenging to combine and analyze the data. Additionally, if the training data is limited or unrepresentative of the broader population, overfitting can occur, resulting in poor generalization of new data, and posing a challenge to the development of AI models for predicting drug metabolism and excretion.

Transparency and interpretability of AI models to predict drug metabolism and excretion are important factors in ensuring the safety and efficacy of these models and their usefulness in clinical applications. However, achieving transparency and interpretability in AI models for drug metabolism and excretion prediction can be challenging, particularly given the complexity of the underlying biological processes. Interpretable AI applications should have desirable features such as transparency, justification, informativeness, and uncertainty estimation [120]. However, many AI models, particularly DL models, are considered “black box” models and are often difficult for human experts to interpret [120,121] because they are highly complex, with multiple layers and nonlinear interactions between different components. Model interpretability is dependent on the chemical representation and AI strategy of choice [122]. Full comprehension of DL models in the context of drug R&D may be challenging to attain, but the supplied predictions might still be helpful to the researcher.

## 7. Conclusions and Future Direction

The development of AI models for drug metabolism and excretion prediction holds great promise for improving drug R&D. Researchers are working hard to explore new ways to create and integrate experimental data, such as relying on metadata to improve data quality [123]. In addition, efforts are underway to improve the quality of experimental data through standardization of experimental protocols, and the use of quality control measures and rigorous validation procedures. The integration of multi-omics data [124], such as genomics, transcription, proteomics, and metabolism, and integration with pharmacokinetic and toxicological modeling will allow for more comprehensive predictions of drug metabolism and excretion. This will allow for a better understanding of the molecular mechanisms underlying ADMET processes and the development of more accurate predictive models. The creation of common data standards and protocols, as well as networks and platforms for sharing data, makes it easier for different institutions and organizations to work together and share data. Collaboration and data sharing among researchers, pharmaceutical companies, and regulatory agencies can aid in the improvement of data quality and availability for AI model development. Model sharing can also help with the validation and testing of AI models in various contexts. AI models for drug metabolism and excretion prediction may be integrated with electronic health records to allow for more personalized medicine by considering individual patient characteristics such as genetic information, age, sex, and medical history. In the context of complex big data, DL methods are likely to prevail soon as they are easier to adapt to a wider range of chemical entities and modeling tasks and enable more efficient data mining. In addition to using existing explanatory AI methods such as feature attribution, instance-based, graph-convolution-based, and self-explanatory methods [123], efforts are being made to develop new methods to ensure transparency, safety, efficacy, and reliability in clinical settings, and maintain public trust in AI technology. In-depth knowledge of drug metabolism and excretion and AI techniques is very important to give a reasonable and useful explanation.

Overall, we provided a comprehensive overview of recent AI-based drug metabolism and excretion prediction research, along with key challenges and future directions. A collaboration effort between AI experts, data scientists, chemists, biologists, and other related field experts and the integration of emerging technologies will be essential to realizing the full potential of this field.

## Data Availability

Not applicable.
